# Restoration of Fertility after Removal of Extrauterine Mirena Coil: A Case Report and Review of the Literature

**DOI:** 10.1155/2011/189565

**Published:** 2011-08-16

**Authors:** Smriti R. C. Bhatta, Radwan Faraj

**Affiliations:** ^1^Sheffield Teaching Hospitals NHS Trust, Sheffield S10 2SF, UK; ^2^Rotherham Hospital, NHS Trust, Rotherham S60 2UD, UK

## Abstract

We present the case of a 27-year-old lady who was seen in the infertility clinic with a history of secondary infertility of a one-year duration. She had a hysteroscopy and Mirena insertion for heavy periods. Coil strings were not found by the GP during first coil check six weeks after insertion. A pelvic ultrasound scan did not show any coil, and it was not investigated further with a possible diagnosis of coil expulsion made. One year following that, she was seen in the infertility clinic. Initial investigations revealed anovulation, and HSG located the coil to be extrauterine. Mirena was removed laparoscopically, and a month following the removal she conceived. She is currently pregnant. This case highlights the effect of extrauterine mirena coils on fertility by possibly causing higher plasma levels of levonorgesterol and resulting suppression of ovulation. Laparoscopic removal of mirena coil can help in restoration of fertility.

## 1. Case Report

We present the case of a 27 year old lady who was seen in the infertility clinic with a history of secondary infertility of a one-year duration. Her BMI was 32. She had two children born by vaginal delivery, and last childbirth was five years ago. Following that she was investigated for heavy periods and stress incontinence and underwent hysteroscopy, Mirena insertion, TVT secure, and posterior repair on the same day. During the first coil check with her general practitioner (GP) 6 weeks after the insertion, the coil strings were not seen and therefore an ultrasound scan was arranged. The Mirena coil was not seen on the scan and was thought to be expelled. No further investigation was arranged.

Approximately a year and a half following that she was seen in the infertility clinic. She gave a history of few months of implanon use for contraception before discontinuing and trying for conception. She had irregular cycles varying from 4- to 8-week intervals and no history of intermenstrual or postcoital bleeding. Her cervical smears were normal and had a prior episode of CIN3 nine years ago treated with LLETZ. There was a history of chlamydia and herpes infections treated in the past. She had a medical problem of ectopic heart beats due to inherited cardiac condition of long QT syndrome for which she had an implantable cardioverter-defibrillator (ICD) inserted five years ago. This was diagnosed following investigations of sudden cardiac death of her brother. She was a nonsmoker and had 18 units of alcohol per week. Her current partner was 34-years-old and was fit and well with no significant past history. He had fathered a child in his previous relationship.

Findings on clinical examination of the couple were normal. The initial investigations done revealed a normal follicular phase, normal serum prolactin, and luteal phase serum progesterone of 28.9 indicating anovulation or borderline ovulation. The semen analysis was normal. A hysterosalpingogram was arranged to check for the tubal patency and it revealed both tubes to be patent and finding of misplaced coil outside the uterus in the abdominal cavity.

The couple were reviewed with the investigation results and the lady was counselled regarding a laparoscopy and retrieval of the extrauterine Mirena coil which she agreed to. She was advised to switch off the tachycardia component of the ICD prior to the operative procedure. During laparoscopy the coil was seen in the pouch of Douglas and no adhesions were noted. The uterus was of normal size and there was evidence of old fundal perforation. Both tubes and ovaries were normal in appearance.

 A followup was arranged in the infertility clinic three months later. Prior to that she confirmed having a positive pregnancy one month after the Mirena coil removal.

She was referred to the high-risk pregnancy clinic for followup in view of having a cardiac defibrillator and previous history of TVT. Her pregnancy is progressing uneventfully and she is currently 25 weeks pregnant.

## 2. Review of the Literature

Intrauterine contraception is a widely used, highly effective method of birth control. Mirena levonorgestrel intrauterine system (LNG IUS) has been licensed in the UK as a contraceptive since May 1995. It is a polymer cylinder mounted on a T-shaped frame and covered with a release rate-controlling membrane which releases 20 microgram/24 h of levonorgestrel. Most outstanding features of LNG IUS are its high contraceptive efficacy and reduction of menstrual blood flow. It is approved for 5-year use. No single mode of action can account for its contraceptive efficacy. The endometrium becomes thin and inactive, and the cervical mucus turns scanty and viscous. Although ovulation may be disturbed to some degree, estradiol production continues normally. After removal of the IUS, normal fertility is regained after a few months, with a near-normal 80% of women able to conceive within 12 months. Same is demonstrated in our case report where the lady achieved conception two months after removal of the Mirena coil.

Misplaced IUS may be asymptomatic, but more commonly, patients complain of irregular bleeding, pelvic pain or cramping, dyspareunia, vaginal discharge, or absent strings. If strings are not visible, the possible explanations could be that IUS is intrauterine with strings broken or coiled in cervix, expulsion which is unnoticed, or uterine perforation and extrauterine migration.

Intrauterine contraceptive device (IUCD) expulsion is a rare event occurring more often in the first year of use in approximately 6% using the LNG IUS or Mirena [1]. A retrospective cohort study [2] compared the dislocation rate of the Multiload 375 IUD (ML 375) and the LNG IUS in 214 women (107 subjects with each IUCD) and found significantly lower number of dislocations in LNG IUS users. Previous expulsion was associated with a significantly higher risk for a reexpulsion in both IUCD groups. There is little evidence concerning risk factors for the expulsion of the LNG IUS. The expulsion rate for the LNG IUS has been reported to be slightly increased in women younger than 25 years [3]. 

An ultrasound should be requested to detect a misplaced IUS and confirm its correct location within the uterus. Accurate ultrasound detection of an IUCD and its position depends on a variety of factors. These include such things as the type of IUCD (copper, hormonal, or inert), the presence of uterine anomalies or scarring, a retroverted uterus, and other patient characteristics that may interfere with image quality [4]. In addition, ultrasound is always operator dependent, and interpretations may vary with the clinical experience of the sonographer. Sonography is also important in assessing the complications of IUCDs, including a low position, associated infection, myometrial migration, uterine perforation, and associated intrauterine or extrauterine pregnancy. If an IUCD is known to be present but not visualized sonographically, plain radiography is helpful in assessing the location. A case report [5] concluded that judicious use of abdominal X-ray can lead to early detection of migrated IUS and expedite removal. Rarely, hysteroscopy and laparoscopy is necessary for localization.

A potentially serious complication is the perforation of the uterus, with reported incidence of 0.5–1/1000 insertions [6]. A study performed in The Netherlands [7] focused on uterine perforations with an LNG IUS and reports an estimated incidence of at least 2.6 per 1000 insertions. This study concluded that perforations are significantly underreported and actual perforation rates are likely higher [7]. The risk of perforation may be increased in lactating women, in women with fixed retroverted uteri, and during the postpartum period. To decrease the risk of perforation postpartum, Mirena insertion should be delayed a minimum of 6 weeks after delivery or until uterine involution is complete [8].

After perforation, devices have been found in various locations in the pelvis and abdomen. Extrauterine migration of an intrauterine device can be life threatening and require emergent surgical intervention and treatment [9]. Delayed detection of perforation may result in migration outside the uterine cavity, adhesions, peritonitis, intestinal perforations, intestinal obstruction, abscesses, and erosion of adjacent viscera. The potential migration of an IUCD and resultant uterine perforation must be considered in the differential diagnosis of any woman using this type of contraception who presents with abdominal pain. More often, they may have a more subtle presentation and have only mild tenderness on examination. Therefore, it is important for the physician to obtain a full history and physical examination and to include these complications on their list of potential diagnoses [8]. It has not been proven that the removal of an asymptomatic displaced IUCD (neither medicated nor nonmedicated) is indicated. Currently, it appears reasonable not to intervene surgically in asymptomatic patients with an intra-abdominal IUD after perforation [10]. This finding is contrary to the recommendation of the Faculty of Sexual and Reproductive Health which advises surgical retrieval of an extrauterine device [10]. It suggests that the symptoms, age, and medical history of the patient must be taken into account, as the benefits of surgery should be balanced against the risks. If the woman is asymptomatic the intra-abdominal IUD can be removed by laparoscopy, in keeping with current guidelines. However, if a laparotomy would be required, consideration should be given to the morbidity and mortality this would cause [11]. Based on our report, we suggest that extrauterine IUS can be a factor implicated in women's abnormal bleeding and may interfere with ovulation. In infertility cases, it would seem very reasonable to perform laparoscopic removal.

A recent study done at Netherlands Pharmacovigilance Centre [12] reported that 8.5% of uterine perforations are detected at the time of insertion. The study concluded that uterine perforations can be asymptomatic and may remain undetected for a long time after IUD insertion. Abdominal pain, control/check-up visits, or changes in bleeding patterns are triggers for detection of perforation and should therefore be taken seriously.

Extrauterine IUD including Mirena and copper coils have the potential to cause adhesions. A study [13] evaluated eight cases of dislocated IUDs and at laparoscopy all disclosed mild local peritoneal adhesions between omentum and pelvic organs. The peritoneal adhesions potential of LNG-IUS was found to be low, similar to that of the copper-bearing IUD and there was no difference in the appearance of the peritoneum in the presence of either a copper IUD or a LNG-IUS. From the authors experience, adhesions is not a common finding.

A previous case report [14] showed finding of an extrauterine IUCD in a women who was being investigated for secondary infertility and in whom there was a history of missing/expulsion of Mirena. This suggests the association of reduced fertility with extrauterine coils, similar to our report.

Another case report published in 2003 [15] and which is relevant to our case study reported that intraperitoneal dislocated LNG-IUS resulted in plasma LNG levels 10-times higher (4.7 nmol/l) than the plasma level of LNG observed with LNG-IUS placed in utero. They measured the LNG and sex hormone-binding globulin plasma concentrations prior to and following the laparoscopic removal. They found that after removal of the device plasma levels drop by 15% to 4 mol/l. The level of levonorgestrel does not have any untoward effects; however, it may continue to suppress ovulation. Conclusion was that the higher levels suppress ovulation, and, therefore, a misplaced LNG-IUS should be removed when pregnancy is desired. Amenorrhoea reported in another case study was also suggestive of this [16].

None of the studies comment on the long-term effects of leaving the device in situ as most are removed laparoscopically [17].

## 3. Conclusion

The levonorgestrel-releasing intrauterine system is a reliable, reversible, low-maintenance method of long-term contraception. Rates of failure are similar to those of female sterilization, and the risk of expulsion is minimal for most users.

A rare but potentially serious complication is that of uterine perforation. This may occur either during the device's insertion or from its later embedment into the uterine wall and subsequent migration through to the intra-abdominal cavity. Perforation can cause internal scarring, infection, or damage to other organs, and may require surgery.

Intraperitoneal dislocated Mirena can cause higher plasma levels of levonorgesterol causing suppression of ovulation and resulting infertility. Removal of Mirena coil as shown in our case report can help in restoration of fertility.

With regards to the limitation of our case report, we did not measure the levonorgestrel level in blood to assess its causal relationship with anovulation. However, achieving conception within 2 months of removal of the Mirena coil supports this association.
